# A Snapshot of Prescribing in Intellectual Disability CAMHS

**DOI:** 10.1192/bjo.2022.491

**Published:** 2022-06-20

**Authors:** Struan Simpson

**Affiliations:** NHS Education for Scotland, Glasgow, United Kingdom

## Abstract

**Aims:**

There is increasing recognition of the use of psychotropic medication in young people with intellectual disability (ID) at a population level but little is known about day-to-day prescribing practice. This project aimed to characterise medication use in this group and assess standards of prescribing practice with reference to RCPsych guidelines.

**Methods:**

Data werecollected by case note review of young people prescribed psychotropic medication within a community ID CAMHS Service. An index prescription was assessed against standards of prescribing - this was the longest standing script for each young person in the study.

**Results:**

73 young people were recruited, aged 7–20 years, predominantly with moderate or severe ID. There was a high degree of comorbidity predominantly with autistic spectrum disorder (ASD), attention deficit hyperactivity disorder (ADHD) and anxiety presentations. Diagnoses did not differ by sex (p > 0.05) however behaviours that challenge were proportionately higher in females (p = 0.014). A high proportion of youngsters displayed behaviours that challenge (68.5%, n = 50) and almost all of these young people (96%, n = 48) had an additional diagnosis. ADHD presentations were negatively associated with behaviours that challenge (p = 0.047).

The hypnotic melatonin was most frequently used medication (56.2%, n = 41) followed by SSRI's (49.3%, n = 36) and antipsychotics (20.5%, n = 15). It was common for use of multiple medications (67.1%, n = 49), typically combining melatonin with a stimulant, SSRI or antipsychotic medication (61%, n = 31). Medications were generally used at modest doses.

The index prescription was in place for a median of 25 months (IQR 28.5, Range 1–108). The indication for prescribing was well documented (98.6%, n = 72) however severity (67.1%, n = 49) and frequency (56.2%, n = 41) recording was poorer. 6-monthly review rate was relatively low (62.5%, n = 40) but the likelihood of review did not reduce with increasing prescription length (p > 0.05). Review of medication response (94.2%, n = 65) and side-effects (73.9%, n = 51) was good. Overall there was poor documentation around consent-to-treatment procedures for young people over 16 years of age with only 17.2% (n = 5) having valid authorisation for medication in their case notes.

**Conclusion:**

This study provides rich clinical data about current clinical practice around prescribing in youngsters with ID. Comorbidity is common and results suggest there may be a bias in labelling behaviours that challenge in males as ADHD-related. A range of (multiple) psychotropic medications are used, often for long-periods despite a lack of evidence base. Clinicians are encouraged to ensure rigorous review and consent-to-treatment processes to minimise harms and over-prescribing in this vulnerable population.

